# Gene Expression in Self-repressing System with Multiple Gene Copies

**DOI:** 10.1007/s11538-013-9808-7

**Published:** 2013-01-25

**Authors:** Jacek Miȩkisz, Paulina Szymańska

**Affiliations:** 1 Institute of Applied Mathematics and Mechanics, University of Warsaw, Banacha 2, 02-097 Warsaw, Poland; 2 College of Inter-Faculty Individual Studies in Mathematics and Natural Sciences, University of Warsaw, Warsaw, Poland

**Keywords:** Self-repressing gene, Multiple gene copies

## Abstract

We analyze a simple model of a self-repressing system with multiple gene copies. Protein molecules may bound to DNA promoters and block their own transcription. We derive analytical expressions for the variance of the number of protein molecules in the stationary state in the self-consistent mean-field approximation. We show that the Fano factor (the variance divided by the mean value) is bigger for the one-gene case than for two gene copies and the difference decreases to zero as frequencies of binding and unbinding increase to infinity.

## Introduction

One of the fundamental processes taking part in living cells is regulation of gene expression. It enables cells to differentiate and adapt to a changing environment. Gene expression is a complex process involving many biochemical reactions with proteins being final products. Produced proteins may in turn enhance or repress expression of other proteins. They may also regulate their own expression. Such regulatory networks in cells, from the smallest ones to those very complicated, have been arousing growing interest recently (Becskei and Serrano 2000; Thattai and van Oudenaarden 2001; Kepler and Elston 2001; Simpson et al. 2003; Lipshtat et al. 2005; Lipniacki et al. 2006; Hat et al. 2007; Komorowski et al. 2009; Loinger and Biham 2009). In many cases, biochemical processes take place in small volumes and may involve only few molecules. Deterministic approach dealing with macroscopic concentrations of molecules (such as ordinary differential equations of classical chemical kinetics) is then inappropriate. A small number of molecules taking part in gene expression results in significant random fluctuations and to take into account such fluctuations, many stochastic models were proposed (Thattai and van Oudenaarden 2001; Swain et al. 2002; Paulsson 2004, 2005).

In many cases, genes exist in several copies (Hat et al. 2007) (and references therein). Understanding the influence of the number of gene copies on the behavior of the system is crucial for designing experiments, which very often involve transfection—introducing an extra copy of the gene with a fluorescent marker in order to observe the evolution of the system. We must take into account that an additional copy of the gene might change the global behavior of the cell. It has been argued in Hat et al. (2007) that the knowledge of how the number of gene copies influences gene expression might lead to a better understanding of experimental data in cancer research. In fact, cancerous cells have, due to mutations, a larger number of gene copies, and thus predictions for tumor’s invaded systems are not the same as for healthy ones.

The minimal model of gene expression, that is, of the production of protein molecules in living cells, consists of four fundamental biochemical processes: transcription (production of mRNA molecules), translation (production of protein molecules), and degradation of molecules of both types. One can compute in this model all moments of the number of protein molecules in the stationary states. In particular, a simple formula for the variance was derived in Thattai and van Oudenaarden (2001); see also Swain et al. (2002), Paulsson (2004, 2005) and Paszek (2007). Here, we lump transcription and translation into one process, that is, we use a standard approximation proposed in Kepler and Elston (2001), which is valid if transcription is much faster than translation.

We analyze a simple model of a self-repressing system with one or two gene copies. Protein molecules may bind to DNA promoters and repress their own transcription. We assume here that each gene copy can be in the unbound state or in the bound state with a lower transcription rate. Such an interaction of protein molecules with transcription factors makes the rigorous analysis of the cell dynamics very difficult. In the case of only one gene copy, exact results were obtained recently in Hornos et al. (2005) and Ramos et al. (2011). In particular, a stationary probability distribution of the number of protein molecules was presented as a series involving Kummer functions (Hornos et al. 2005). Time evolution of the probability distribution was considered in Ramos et al. (2011).

Here, we obtain explicit formulas for the variance of the number of protein molecules in the stationary state in the self-consistent mean-field approximation. Such approach, used commonly in statistical physics (Huang 1963; Ma 1985), was introduced recently in gene expression models in Ohkubo (2010); see Miȩkisz and Szymańska (2012) to compare a mean-field approximation in the Ising model of interacting spins and in a simple model of self-repressing gene. We also discuss two extreme cases: slow switching (binding/unbinding), where to get analytic results we can use the conditional variance or simply perform an appropriate limit and fast gene switching, where we use an adiabatic approximation. We show analytically that in both extreme cases, the stationary variance of the number of protein molecules coincides with the mean-field approximation. We solved a truncated system of Master equations and showed that the solution agrees with the mean-field approximation for the whole range of the adiabaticity parameter.

The main goal of this paper is to establish how the number of gene copies influences the variance of produced proteins in a simple case of a self-repressing gene. We show that the two-gene system has a lower Fano factor (the variance divided by the mean value than the one-gene regulatory system). The difference disappears when the rate of switching becomes large as compared to production and degradation rates, that is in the adiabatic limit.

In Sect. 2, we analyze one-gene model. Two gene copies are discussed in Sect. 3. Section 4 is devoted to the fast switching gene case, and Sect. 5 to the slow switching one. Conclusions follow in Sect. 6.

## Self-repressing Gene

Here, we analyze the simplest model of a self-regulating gene. We lump transcription and translation into one process, so we assume that proteins are produced directly out of DNA in one biochemical process (Kepler and Elston 2001). We will discuss here the repression—protein molecules may bind to a certain promoter region of their own DNA, and thus decrease or completely stop the transcription. In continuous models of chemical kinetic equations, the repression is modeled by the modification of a transcription rate, it might be given by a Hill function *h*(*n*)=*k*/(1+*cn* ^*h*^), where *k* is the maximal transcription rate, *n* the number of repressing protein molecules, *c* and *h* are constants (Komorowski et al. 2009).

**Fig. 1:**
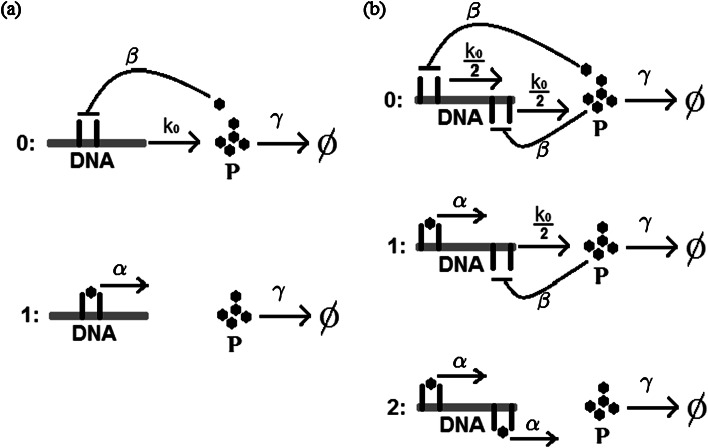
(**a**) One-gene system. The protein is produced directly from the DNA in state 0 with the rate *k*_0_, a single molecule degrades with rate *γ*. The production is switched off when a protein binds to the promoter region. Binding and unbinding takes place with *βn* and *α* rates, respectively. (**b**) Two-gene system. Molecules bind to promoters of both gene copies independently

Here, we will consider a stochastic model, where the gene (DNA) can be in two discrete states: unbound (on), denoted by 0 or bound (off), denoted by 1. In the generic case, the transcription rates for the on- and off-states are given by *k*_0_ and *k*_1_ respectively, but we set *k*_1_=0, as it is often done. The protein degradation rate is denoted by *γ*. We consider a monomer binding and thus we assume that the binding rate is given by *βn*, where *n* is the number of proteins in the system, and the rate of switching the gene on (unbinding) is denoted by *α*; see Fig. 1.

Let us introduce formally our model. We denote by *f*_*i*_(*n*,*t*), *i*=0,1 the joint probability that there are *n* protein molecules in the system at time *t* and the gene (DNA) is in the state *i*. The standard Master equation (Van Kampen 1997) can be written as:

1 $$ \begin{aligned}[c] \frac{d}{dt}f_{0}(n,t) &= k_{0} \bigl[f_{0}(n-1)-f_{0}(n)\bigr]+ \gamma\bigl[(n+1)f_{0}(n+1)-nf_{0}(n)\bigr] \\ &\quad- \beta nf_{0}(n)+\alpha f_{1}(n) \\ \frac{d}{dt}f_{1}(n,t)& = k_{1}\bigl[f_{1}(n-1)-f_{1}(n) \bigr] + \gamma\bigl[nf_{1}(n+1)-(n-1)f_{1}(n)\bigr] \\ &\quad+ \beta nf_{0}(n)-\alpha f_{1}(n) \end{aligned} $$

for *n*≥1.

For *n*=0 we have $\frac{d}{dt}f_{0}(0,t)= -k_{0}f_{0}(0)+\gamma f_{0}(1)$ and *f*_1_(0,*t*)=0.

Let us emphasize that *n* is the total number of molecules; one of them is bound to the promoter when the gene state is 1. It follows that *f*_1_(0,*t*)=0 all the time. We have also assumed that the bound protein cannot degrade. In this respect, our Master equation is different from the one discussed in Hornos et al. (2005); see also Qian et al. (2009).

We denote by (*f*_0_,*f*_1_) a stationary state of our system, that is a solution of (1) with time derivatives set to zero. Let *A*_0_ and *A*_1_ be probabilities (frequencies) that the gene is unbound or bound, respectively, in the stationary state, $A_{i} = \sum_{n=0}^{+\infty}f_{i}(n),\ i=0,1$. The stationary expected number of protein molecules with respect to *f*_*i*_ is given by $\langle n\rangle_{i}= \sum_{n=0}^{+\infty}nf_{i}(n)$, obviously 〈*n*〉=〈*n*〉_0_+〈*n*〉_1_ is the expected value with respect to *f*=*f*_0_+*f*_1_. We introduce two generating functions: 
 We differentiate generating functions with respect to time, use (1), and after some simplifications, we get 
2 Now we differentiate the above equations with respect to *z* once and twice, set *z*=1, time derivatives to zero, and get the following algebraic equations for the moments of the stationary probability distribution of the number of protein molecules: 
3


The above system is hierarchical, equations for lower moments involve higher moments (unlike equations in the classical model of unregulated gene expression analyzed in Thattai and van Oudenaarden 2001). It is not closed (there are more variables than equations) and, therefore, in principle cannot be solved. In order to get explicit formulas for moments, in particular the variance, one has to close somehow the infinite chain of equations. Several concepts and techniques were developed (Nasell 2003; Barzel and Biham 2011; Barzel et al. 2011). Here, we will use the so-called mean-field approximation well known in statistical physics of interacting particles (Huang 1963; Ma 1985; Miȩkisz and Szymańska 2012) and introduced recently in the context of regulatory genetic systems in Ohkubo (2010). Namely, we replace *n* in the switching term in (1) by its unknown expected value, that is instead of *βnf*_0_(*n*) we write $\beta\frac{\langle n\rangle_{0}}{A_{0}}f_{0}(n)$. It follows that (3) is replaced by

4 $$ \begin{cases} A_{0}+A_{1}=1\\ \beta\langle n\rangle_{0}-\alpha A_{1}=0\\ \noalign{\vspace{3pt}} k_{0}A_{0} - \gamma\langle n\rangle_{0} -\beta\frac{\langle n\rangle _{0}}{A_{0}}\langle n\rangle_{0}+\alpha\langle n\rangle_{1}=0\\ \noalign{\vspace{3pt}} k_{1}A_{1} -\gamma\langle n\rangle_{1}+\gamma A_{1} + \beta\frac {\langle n\rangle_{0}}{A_{0}}\langle n\rangle_{0}-\alpha\langle n\rangle _{1}=0\\ \noalign{\vspace{3pt}} 2k_{0}\langle n\rangle_{0} - 2\gamma\langle n(n-1)\rangle_{0} - \beta \frac{\langle n\rangle_{0}}{A_{0}}\langle n(n-1)\rangle_{0} +\alpha \langle n(n-1)\rangle_{1}=0\\ \noalign{\vspace{3pt}} 2k_{1}\langle n \rangle_{1} -2\gamma\langle n(n-1)\rangle_{1} +2\gamma (\langle n\rangle_{1}-A_{1})+ \beta\frac{\langle n\rangle _{0}}{A_{0}}\langle n(n-1)\rangle_{0} \\ \noalign{\vspace{3pt}} \quad-\alpha\langle n(n-1)\rangle_{1}=0 \end{cases} $$

We obtained a closed system of equations. Let us observe that when one adds the third equation and the fourth one of either (3) or (4), results are the same (switching terms cancel out). The same applies to adding the fifth equation and the sixth one. Hence, independent of approximations, the following relations are always satisfied:

5 $$ \begin{cases} \langle n \rangle= \frac{k_{0}}{\gamma}A_{0} + \frac{k_{1}}{\gamma }A_{1} + A_{1}\\ \noalign{\vspace{3pt}} \langle n(n-1)\rangle= \frac{k_{0}}{\gamma}\langle n\rangle_{0} + \frac {k_{1}}{\gamma}\langle n\rangle_{1} + \langle n \rangle_{1} - A_{1} \end{cases} $$

One can solve (4) (in fact we only need to solve first four equations), obtain the self-consistent value for 〈*n*〉_*i*_,*i*=0,1, use (5) and var(*n*)=〈*n*(*n*−1)〉+〈*n*〉−〈*n*〉^2^ to get the expression for the variance of the number of protein molecules in the stationary state.

Here, we set *k*_1_=0 and following Hornos et al. (2005) introduce new parameters: $X^{\mathrm{eq}}=\frac{\alpha}{\beta}$—equilibrium constant of the switching process, $X^{\mathrm{ad}}=\frac{k_{0}+k_{1}}{2\gamma}=\frac{k_{0}}{2\gamma }$—measure of protein concentration, and $\omega=\frac{\alpha}{\gamma }$—adiabaticity parameter. It appears that all equations can be written in terms of these parameters.

From the first four equations of (4), we get the quadratic equation for *A*_1_, 
6 which has only one positive solution smaller than 1.

Equation (4) allows us to express var(*n*) as a function of *A*_1_, 
7


**Fig. 2:**
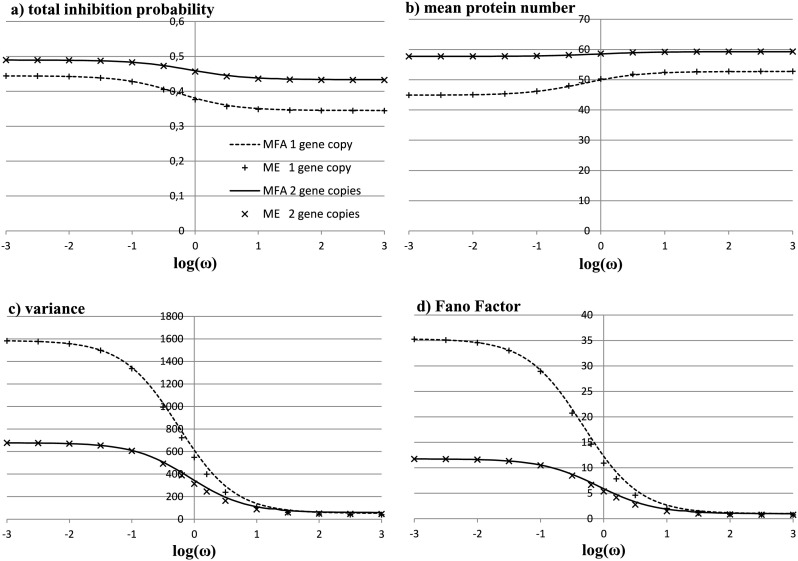
Total inhibition probability, mean, variance, and Fano Factor of the number of protein molecules in the stationary state, plotted as a function of log(*ω*) for *X* ^eq^=100 and *X* ^ad^=40. For the variance, the curves asymptotically approach values computed in the slow-switching case (1585 for the one-gene model and 678 for two gene copies) and in the adiabatic case (52 and 59 for the one and two-gene model, respectively). *Continuous* and *dotted lines* are obtained analytically within the mean-field approximation, crosses and pluses are points obtained by solving the system of Master equations (Eqs. (1) and (8)) restricted to the maximum of 200 particles

The variance as a function of log*ω* is presented in Fig. 2. We see that the variance is a decreasing function of the switching rate.

We would like to check the validity of the mean-field approximation in two extreme cases: in the limits of the infinitely fast and infinitely slow switching. In the fast-switching case, we divide equations in (3) by *α* and assume that $\frac{k_{i}}{\alpha}=\frac{\gamma }{\alpha}=0$. However, this does not help us in closing the system (3), the number of equations is still too small. It is usually assumed, for example, in Hornos et al. (2005) that in the fast switching case, in the so-called adiabatic limit, one may put 〈*n*〉_*i*_=*A*_*i*_〈*n*〉, *i*=0,1. Such a procedure closes (3). We would like to point out however that this is another approximation and it is not true even in the limit *α*,*β*→∞; see Sect. 4. In the slow-switching case, we assume that for a given gene state, the system attains its stationary state (if *k*_1_=0, then of course all protein molecules are degraded in the stationary state). In such stationary states, we have from Thattai and van Oudenaarden (2001) formulas for the variance even in the model with transcription and translation; in our simplified model stationary states have the Poisson distribution and so the variance is equal to the expected value. Then we take into account switching between gene states—we simply use the conditional variance formula; see Sect. 5. Alternatively, we may close (3) by dividing equations by *k*_0_ and assuming that $\frac{\alpha}{k_{0}} = \frac{\beta }{k_{0}} =0$, details are shown in Sect. 5. We see in Fig. 2 that the mean field-approximation coincides with the fast-switching solution in the limit of the infinite *ω* and with the slow-switching one in the limit of zero *ω*.

To validate the mean-field approximation, we truncated the Master equation (1) by restricting the number of protein molecules to be at most 200. The rigorous solution of the truncated Master equation agrees with the mean-field solution for the whole range of the adiabaticity parameter *ω* as it can be seen in Fig. 2.

## Repression with Two Gene Copies

**Fig. 3:**
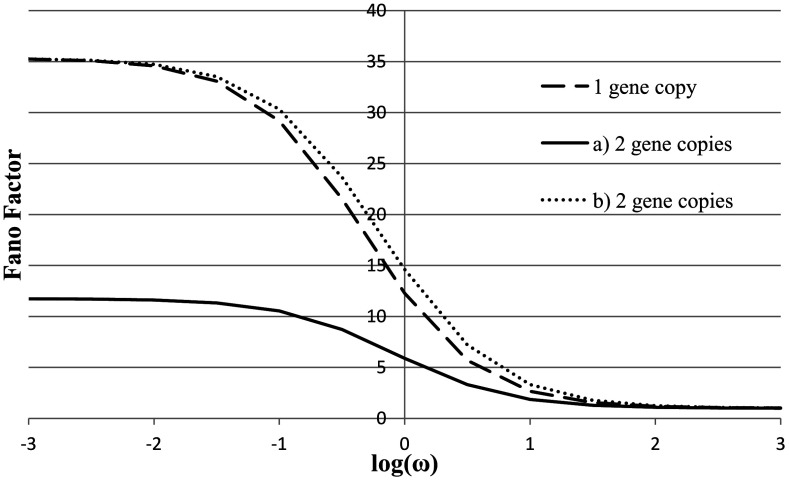
Fano factor as a function of log(*ω*), *X* ^eq^=100 and *X* ^ad^=40 for one-gene model, (**a**) for two-gene model with the production rate *k*_0_/2 (**b**) for two-gene model with the production rate *k*_0_

Now we assume that the gene is present in two copies. It follows that the gene system can be in three states: 0, 1, and 2, where 0 means that both promoter sites are unbound, 1 means that exactly one promoter is bound, and 2 that both promoters are bound. Both copies of the gene produce proteins independently. To keep the mean expression approximately at the same level as in the one-gene case, we set $k_{1}=\frac{k_{0}}{2}$ and *k*_2_=0 so *X*_ad_=(*k*_0_+*k*_1_+*k*_2_)/3*γ*=*k*_0_/2*γ* as before. That is we assume that production rates of both genes are set to $\frac{k_{0}}{2}$. We also made calculations for the production rates of two genes equal to *k*_0_, they are literally copies of original genes. The mean and the variance are then approximately doubled, but the Fano factor (the variance divided by the mean) remains the same; see Fig. 3.

The Master equation now reads: 
8 for *n*≥2 and we may write similar equations for *n*=1 and *n*=0 with obvious terms not present.

We replace *n* in the switching term in (8) by its unknown expected value, that is instead of *βnf*_0_(*n*) and *βnf*_1_(*n*) we write $\beta\frac{\langle n\rangle_{0}}{A_{0}}f_{0}(n)$ and $\beta\frac{\langle n\rangle_{1}}{A_{1}}f_{1}(n)$ respectively. We introduce three generating functions, repeat the procedure of the previous section, and get a closed system of equations in the mean-field approximation,

9 $$ \begin{cases} A_{0}+A_{1}+A_{2}=1\\ \beta\langle n\rangle_{0}-\alpha A_{1}=0\\ \beta\langle n\rangle_{1}-2\alpha A_{2} - \beta A_{1}=0\\ \noalign{\vspace{3pt}} k_{0} A_{0} - \gamma\langle n\rangle_{0}- \beta\frac{\langle n\rangle ^{2}_{0}}{A_{0}}+ \alpha\langle n\rangle_{1}=0\\ \noalign{\vspace{3pt}} (\frac{1}{2}k_{0}+\gamma)A_{1} - \gamma\langle n\rangle_{1} + \beta \frac{\langle n\rangle^{2}_{0}}{A_{0}} - \alpha\langle n\rangle_{1} -\beta\frac{\langle n\rangle^{2}_{1}}{A_{1}} + \beta\langle n\rangle_{1} +2\alpha\langle n\rangle_{2}=0\\ \noalign{\vspace{3pt}} 2\gamma A_{2}-\gamma\langle n\rangle_{2}+\beta\frac{\langle n\rangle ^{2}_{1}}{A_{1}} - \beta\langle n\rangle_{1}-2\alpha\langle n\rangle _{2} =0\\ \noalign{\vspace{3pt}} 2k_{0}\langle n\rangle_{0} - 2\gamma\langle n(n-1)\rangle_{0} - \beta \frac{\langle n\rangle_{0}}{A_{0}}\langle n(n-1)\rangle_{0} +\alpha \langle n(n-1)\rangle_{1}=0\\ \noalign{\vspace{3pt}} (k_{0}+2\gamma)\langle n\rangle_{1}-2\gamma A_{1}- 2\gamma\langle n(n-1)\rangle_{1}+\beta\frac{\langle n\rangle_{0}}{A_{0}}\langle n(n-1)\rangle_{0}\\ \noalign{\vspace{3pt}} \quad-\alpha\langle n(n-1)\rangle_{1}-(\beta\frac{\langle n\rangle _{1}}{A_{1}}-\beta)\langle n(n-1)\rangle_{1}+2\alpha\langle n(n-1)\rangle_{2}=0\\ \noalign{\vspace{3pt}} 4\gamma\langle n\rangle_{2}-4\gamma A_{2} -2\gamma\langle n(n-1)\rangle _{2}\\ \noalign{\vspace{3pt}} \quad+ (\beta\frac{\langle n\rangle_{1}}{A_{1}}-\beta)\langle n(n-1)\rangle _{1})-2\alpha\langle n(n-1)\rangle_{2}=0 \end{cases} $$

We add the fourth equation, the fifth, and the sixth one of (9) and then the last three equations of (9) and again as in the one-gene case we get relations which are satisfied independent of approximations:

10 $$ \begin{cases} \langle n\rangle= \frac{k_{0}}{\gamma}A_{0} + \frac{k_{0}}{2\gamma }A_{1} + 2A_{2} + A_{1} \\ \noalign{\vspace{3pt}} \langle n(n-1) \rangle= \frac{k_{0}\langle n\rangle_{0}+\frac {1}{2}k_{0}\langle n\rangle_{1}}{\gamma}+2\langle n \rangle _{2}+\langle n \rangle_{1}-2A_{2}-A_{1} \end{cases} $$

**Fig. 4:**
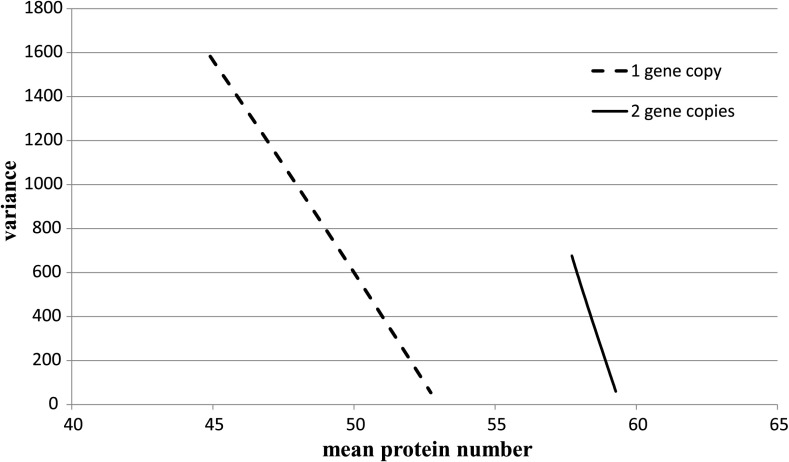
Variance as a function of the mean, *X* ^eq^=100 and *X* ^ad^=40, so that *ω* is the only changing parameter, −3<log(*ω*)<3

As in the one-gene case, all equations can be expressed in terms of $X^{\mathrm{eq}}=\frac{\alpha}{\beta}$, $X^{\mathrm{ad}}=\frac{k_{0}+k_{1}+k_{2}}{3\gamma}=\frac {k_{0}}{2\gamma }$, and $\omega=\frac{\alpha}{\gamma}$. We proceed exactly in the same way as in the one-gene case. We solve the system (9) and get an expression for the probability of total inhibition, the expected value of the number of produced proteins, the variance, and the Fano factor as functions of log(*ω*) in the stationary state; see Fig. 2. We see that the variance and the Fano factor are bigger for the one-gene case than for the two-gene case and that the difference decreases to zero as the rates of gene switching increase. In Fig. 4, we graph the variance as the function of the expected value of the number of proteins as we vary the adiabaticity parameter *ω* while keeping *X* ^eq^ and *X* ^ad^ fixed. We observe the linear dependence, the slope is bigger in the two-gene case than in the one-gene case.

## Fast Switching Gene

Here, we consider the situation when gene states are switched infinitely fast. For simplicity, we discuss one-gene case. Let us assume for a moment that there is no self-regulation and the gene is switched between its two states with constant rates: from the state 1 to the state 0 with the rate *α* and from 0 to 1 with the rate *β* (not *βn* with *n* being the number of protein molecules as in the self-regulating gene case). We will show (as it might be expected) that the expected value of the number of protein molecules in a given state is equal to the expected value of the number of molecules times the frequency of that state, that is 〈*n*〉_*i*_=*A*_*i*_〈*n*〉; *i*=0,1. As in Sect. 2, *f*_*i*_(*n*) are probabilities that there are *n* protein molecules in the system and the gene is in the state *i*. Now instead of (1), we have the following Master equation (we do not assume here that *k*_1_=0):

11 $$ \begin{aligned}[c] \frac{d}{dt}f_{0}(n,t)&= k_{0} \bigl[f_{0}(n-1)-f_{0}(n)\bigr] \\ &\quad+\gamma\bigl[(n+1)f_{0}(n+1)-nf_{0}(n)\bigr] - \beta f_{0}(n)+\alpha f_{1}(n) \\ \frac{d}{dt}f_{1}(n,t) &= k_{1}\bigl[f_{0}(n-1)-f_{0}(n) \bigr] \\ &\quad+\gamma\bigl[(n+1)f_{1}(n+1)-(n)f_{1}n\bigr]\beta f_{0}(n)-\alpha f_{1}(n) \end{aligned} $$

The equations for generating functions (see Sect. 2) now read

12 $$ \begin{aligned}[c] &\frac{\partial F_{0}(z,t)}{\partial t}=(z-1)\biggl[k_{0} F_{0}(z,t) - \gamma\frac{\partial F_{0}(z,t)}{\partial z}\biggr] - \beta F_{0}(z,t) + \alpha F_{1}(z,t) \\ &\frac{\partial F_{1}(z,t)}{\partial t}=(z-1)\biggl[k_{1} F_{1}(z,t) - \gamma \frac{\partial F_{1}(z,t)}{\partial z}\biggr] + \beta F_{0}(z,t) - \alpha F_{1}(z,t) \end{aligned} $$

As in Sect. 2, we differentiate the above equations with respect to *z* once and twice, set *z*=1, time derivatives to zero, and get the following algebraic equations for the moments of the stationary distributions of the number of protein molecules:

13 $$ \begin{cases} A_{0}+A_{1}=1\\ \beta\langle n\rangle_{0}-\alpha A_{1}=0\\ k_{0}A_{0} - \gamma\langle n\rangle_{0} - \beta\langle n\rangle _{0}+\alpha\langle n\rangle_{1}=0\\ k_{1}A_{1} - \gamma\langle n\rangle_{1} + \beta\langle n\rangle _{0}-\alpha\langle n\rangle_{1}=0\\ 2k_{0}\langle n\rangle_{0} - 2\gamma\langle n(n-1)\rangle_{0} - \beta \langle n(n-1)\rangle_{0} +\alpha\langle n(n-1)\rangle_{1}=0\\ 2k_{1}\langle n\rangle_{1} - 2\gamma\langle n(n-1)\rangle_{1} + \beta \langle n(n-1)\rangle_{0} -\alpha\langle n(n-1)\rangle_{1}=0 \end{cases} $$

The above system of equations is closed and it can be solved. In particular, we get $A_{0}=\frac{\alpha}{\alpha+\beta}$ and $A_{1}=\frac{\beta}{\alpha+\beta}$ (this of course follows immediately from the assumption about constant switching rates) and 
14
15 In the limit of infinitely fast switching, that is when $\frac {k_{0}, \gamma}{\alpha, \beta} \rightarrow0$, it follows that 〈*n*〉_0_=*A*_0_〈*n*〉 and then 〈*n*〉_1_=*A*_1_〈*n*〉. The two-gene case and in general *n*-gene case can be treated in the same way and the same conclusion follows.

Gene expression models with constant switching rates were discussed in Paulsson (2004, 2005) and Paszek (2007) and formulas for the variance of the number of protein molecules in the stationary state were derived.

Now we discuss self-repressing genes. It is suggested in Hornos et al. (2005) that also in this case, 〈*n*〉_*i*_=*A*_*i*_〈*n*〉 in the limit of infinitely fast switching. Let us examine this. The second line in (3) reads

16 $$ A_{1}= \frac{\beta\langle n\rangle_{0}}{\alpha} $$

It might also be written as

17 $$ A_{1}= \frac{\beta\langle n\rangle_{0}}{\alpha A_{0} + \alpha A_{1}}=\frac{\beta\langle n\rangle_{0}}{\alpha A_{0} + \beta\langle n\rangle_{0}} $$

It is easy to see that 〈*n*〉_0_=*A*_0_〈*n*〉 is equivalent to

18 $$ A_{1}= \frac{\beta\langle n\rangle}{\alpha+ \beta \langle n\rangle} $$

which is the equilibrium mass action law as discussed in Hornos et al. (2005). However, in the limit of infinitely fast switching, for any fixed *n*, the gene state is in equilibrium, and hence

19 $$ A_{1}= \frac{\beta n}{\alpha+ \beta n} $$

In the stationary state, we have to average the above expression, and we get

20 $$ A_{1}= \biggl\langle\frac{\beta n}{\alpha+ \beta n}\biggr\rangle $$

which in general is different from (18). We have also considered a simple cut-off system with maximally two protein molecules allowed. In such a case one can get analytical formulas for the stationary probability distribution. It appeared that in the adiabatic limit, 〈*n*〉_*i*_≠*A*_*i*_〈*n*〉 but we are very close to the equality. Numerical calculations of the exact, but not explicit formula presented in Hornos et al. (2005) indicate that 〈*n*〉_*i*_=*A*_*i*_〈*n*〉; *i*=0,1 is a very good approximation.

Now we set 〈*n*〉_*i*_=*A*_*i*_〈*n*〉; *i*=0,1. This closes (3) for the one-gene case and the analogous system of equations in the two-gene case. We see in Fig. 2 that in the fast-switching case, the mean-field and adiabatic approximations practically coincide. We will now show how far is the variance from the mean in the adiabatic approximation.

In the one-gene case, (5) together with 〈*n*〉_*i*_=*A*_*i*_〈*n*〉; *i*=0,1 give us

21 $$ \mathrm{var}(n) = \langle n \rangle- A_{1} $$

For the two-gene case, from (10) it follows that

22 $$ \mathrm{var}(n) = \langle n \rangle- A_{1} -2A_{2} $$

We can also get that for large mean expression levels, when one may neglect one protein molecule bound to the promoter, in the adiabatic limit var(*n*)=〈*n*〉.

## Slow Switching Gene

In the slow-switching case, we divide (3) by *k*_0_ and *k*_1_, respectively, and assume that $\frac{\alpha}{k_{i}}=\frac {\beta }{k_{i}}=0$ and get

23 $$ \begin{cases} \langle n\rangle_{0}=\frac{k_{0}}{\gamma}A_{0} \\ \noalign{\vspace{3pt}} \langle n\rangle_{1}=\frac{k_{1}}{\gamma}A_{1} + A_{1}\\ \noalign{\vspace{3pt}} \langle n(n-1)\rangle_{0}=\frac{k_{0}}{\gamma}\langle n \rangle _{0}\\ \noalign{\vspace{3pt}} \langle n(n-1)\rangle_{1}=\frac{k_{1}}{\gamma}\langle n \rangle_{1} + \langle n\rangle_{1} - A_{1} \end{cases} $$

The formula for the variance takes the following form: 
24 Now we use the conditional variance formula

25 $$ \mathrm{Var}(X) = \mathrm{Var}\bigl(E(X|Y)\bigr) + E\bigl(\mathrm{Var}(X|Y)\bigr), $$

where *X* is the random variable describing the number of protein molecules and *Y* describes the gene state. For a fixed state of the gene, *Y*=*i*, the stationary state of production and degradation processes is Poissonian and, therefore, $\mathrm{Var}(X|Y=0) = E(X|Y=0) = \frac{k_{0}}{\gamma }$ and $\mathrm{Var}(X|Y=1) = \frac{k_{i}}{\gamma}$, $E(X|Y=1) = \frac {k_{i}}{\gamma}+1$. It is easy to see that we get exactly the same formula as (24).

The approximation of slow switching has been also used in Qian et al. (2009), but only for the one-gene case. It was of course assumed that when the binding and unbinding rates approach 0, we have two Poisson distributions for the unbound and bound states that we may plug into the Master equation and calculate the total probability that there are *n* proteins in the system.

## Discussion

We analyzed analytically a simple model of a self-repressing system with one and two gene copies. We showed that the stationary variance and the Fano factor are bigger for the one-gene case than for the two-gene case, and the difference decreases to zero as switching rates increase.

We derived our formulas within the self-consistent mean-field approximation. The approximation was tested in two extreme cases: fast switching and slow switching genes. We discussed the validity of the adiabatic approximation for fast switching genes and showed that both mean-field and adiabatic approximations agree in this regime. In the slow-switching case, we derived rigorous formulas, which coincide with the mean-field approximation formulas.

We also established the linear dependence of the variance with respect to the mean as the adiabaticity parameter increases; the slope is bigger in the two-gene case than in the one-gene case.

It would be interesting to use mean-field approximation in other regulatory gene systems, like the toggle switch, and in general in systems with bistabilities.
